# Connexin 31.9 is expressed in human pancreatic endocrine beta cells, as well as in exocrine ductal epithelial and vascular endothelial cells

**DOI:** 10.1042/BSR20250223

**Published:** 2026-08-12

**Authors:** Elia Martha Pérez-Armendariz, Oscar J. Zacarías-Lara, Cristina Revilla-Monsalve, Enrique R. Page-Pérez, Beatriz Hernández-Téllez, Ángel Zarain-Herzberg, César A. Flores-García, Rocío Arreola-Rosales, Marco E. Gudiño-Zayas

**Affiliations:** 1 Laboratorio de Sinapsis Eléctricas, Unidad de Investigación en Medicina Experimental, Facultad de Medicina, UNAM, Universidad Nacional Autónoma de México, Av. Universidad 3000 SN, Colonia UNAM, CU, Ciudad de México 04510, México; 2 Unidad de Investigación Médica en Enfermedades Metabólicas, Unidad Médica de Alta Especialidad, Hospital de Cardiología, Centro Médico Nacional Siglo XXI, Instituto Mexicano del Seguro Social, Ciudad de México 06720, México; 3 Departamento de Biología Celular, Facultad de Medicina, Universidad Nacional Autónoma de México, Av. Universidad 3000 SN, Colonia UNAM, CU, Ciudad de México, 04510, Ciudad de México, 04510, México; 4 Departamento de Bioquímica, Facultad de Medicina, Universidad Nacional Autónoma de México, Av. Universidad 3000 SN, Colonia UNAM, CU, Ciudad de México 04510, México; 5 Servicio de Patología, Unidad Médica de Alta Especialidad, Hospital de Cardiología, Centro Médico Nacional Siglo XXI, Instituto Mexicano del Seguro Social, Ciudad de México,06720, México; 6 Servicio de Patología, Unidad Médica de Alta Especialidad, Hospital de Especialidades, Centro Médico Nacional Siglo XXI, Instituto Mexicano del Seguro Social, Ciudad de México, 06720, México; 7 Laboratorio APADI, Unidad de Medicina Experimental, Facultad de Medicina, Universidad Nacional Autónoma de México, México City, 6720, México

**Keywords:** connexin 30.2, Connexin 31.9, Connexin 36, diabetes, human pancreas, insulin secretion

## Abstract

Connexin 36 (Cx36)-deficient mice exhibit deregulation of insulin secretion and glucose homeostasis. Mouse islet beta cells express Cx36 and connexin 30.2 (Cx30.2). Human beta cells express Cx36; however, it remains unknown whether they express Cx31.9 protein, the ortholog of Cx30.2. To address this question, sections of 23 human pancreatic autopsy samples from both sexes were analyzed by real-time qRT-PCR with oligonucleotides against Cx31.9 and by immunohistochemistry (IHC) with a specific antibody against this connexin. Mean levels of Cx31.9 mRNA were similar to those of Cx36 mRNA, used as a control gene, in all human samples analyzed (*n* = 7). IHC studies showed that Cx31.9-positive staining was localized to most islet cells in donor samples (15 of 16). The density of Cx31.9 staining per μm^2^ was calculated from 31 islet images from 10 donor samples. Pancreatic serial sections revealed that the spatial distribution of anti-Cx31.9-labeled cells is similar to that of anti-Cx36- and anti-insulin-positive cells. Moreover, anti-Cx31.9 staining was also localized in anti-CK19-positive epithelial ductal cells and in the endothelial cells of small, medium, and large-diameter vessels. Cx31.9 protein is expressed in endocrine islet beta cells as well as in exocrine ductal epithelial and vascular endothelial cells. In addition, there is a balance of Cx31.9 and Cx36 mRNA levels in the human pancreas.

## Introduction

Diabetes has been regarded as a pandemic and one of the leading causes of death in many countries in recent decades. According to the International Diabetes Federation, 589 million people live with diabetes, and 90% of them have type 2 diabetes [1]. Insulin is the only hormone that reduces blood glucose levels, making it essential to improve our understanding of the mechanisms that regulate its production and release. Growing evidence indicates that direct intercellular communication through gap junction channels is a new mechanism regulating insulin secretion [2–4].

Direct intercellular communication in vertebrate cells is mediated by connexins (Cxs), a family of related proteins (21 in humans and 20 in mice) that form intercellular channels and membrane hemichannels. Two connexons, each supplied by a cell in an adjacent pair, come together to form an intercellular channel. Each connexon consists of six Cx subunits. Cells typically express more than one Cx [5], and functional homomeric, heterotypic, and heteromeric intercellular channels have been observed in cell expression systems, significantly increasing their molecular diversity and functional capabilities. This diversity is essential for the selective and targeted movement of ions and other messenger molecules between specific cell types in different organs. Additionally, Cx mutations have been linked to human diseases [6].

A high rate of electrical coupling (95%) was identified by recording junctional currents with a tiny unitary conductance (mean γ_j_ 6 pS) in pairs of freshly isolated, double-voltage-clamped mouse beta cells [7,8], similar to that reported for Cx36 channels recorded in cell expression systems [9]. Consistently, Cx36 protein and mRNA have been identified in islet cells [7,10,11]. Furthermore, a reduction in dye transfer between cells in isolated islets [12], as well as a loss of synchronized intercellular [Ca^2+^]_i_ oscillations and pulsatile insulin secretion induced by glucose, was observed in isolated islets from Cx36-deficient mice [13]. Additionally, Cx36-deficient mice showed increased fasting insulin levels [14] and an impaired glucose tolerance curve [15]. Similarly, down-regulation of Cx36 mRNA and protein levels has been found in prediabetic mice [16] and in INS-1 cells cultured in high-glucose medium for 24 h [10]. Therefore, Cx36 is regarded as a key molecule for insulin secretion and glucose homeostasis [3,16,17] and has been considered, for over two decades, the sole connexin expressed in islet beta cells.

Nonetheless, Cx31.9 also forms intercellular channels with a small unitary conductance (mean γj 10 pS) [18], which is at the lower end of those formed by Cx36 (mean γj 10–15 pS) as recorded in a cell expression system [9,19]. In islet beta cells, the expression of Cx30.2 was indicated by their junctional current properties, showing that their intercellular channels have a smaller unitary conductance (mean γj of 6 pS) and a different pharmacological response [7] compared with those of Cx36 intercellular channels [9,19]. Consistently, we identified Cx30.2 mRNA and protein in isolated, cultured mouse islet preparations using quantitative reverse transcription polymerase chain reaction (qRT-PCR) and Western blot analysis [20]. Additionally, we observed that at pancreatic islet beta cell membrane appositions, Cx30.2-immunofluorescent-positive staining overlaps with that of Cx36. Furthermore, we found that islets cultured in high glucose decrease their Cx30.2 mRNA levels [20]. Similarly, mouse retinal cells cultured in high glucose also show decreased Cx30.2 mRNA levels [21].

Additionally, in the mouse pancreas, Cx30.2 was identified in ductal exocrine epithelial and vascular endothelial cells [22]. Therefore, it is essential to determine whether Cx31.9 protein, the ortholog of Cx30.2, is expressed in the human pancreas. In this regard, human islet beta cells respond to glucose stimulation with [Ca^2+^]_i_ oscillations [23] and express the Cx36 protein [17]. Additionally, a band corresponding to the expected molecular weight of Cx31.9 mRNA was detected in total human pancreas preparations by Northern blot analysis [24] and in isolated islets by multi-Cx analysis using endpoint PCR [25]. However, it remains unknown whether the Cx31.9 protein is expressed in human islet cells and, if so, what its cell-specific distribution is.

In the present study, we examined these questions by performing immunohistochemical analyses of paraffin-embedded necropsy sections from human subjects of both sexes and by detecting Cx31.9 mRNA using qRT-PCR. Our results demonstrate that there is a balance between Cx31.9 and Cx36 mRNA levels in the human pancreas and that Cx31.9 protein is localized to pancreatic beta cells. Additionally, Cx31.9 is also expressed in the exocrine component of the pancreas in ductal epithelial and vascular endothelial cells.

## Materials and methods

### Pancreatic human samples

Human autopsy samples were obtained from paraffin-embedded necropsy specimens provided by the Pathology Unit at the Cardiology and Specialities Hospitals of the Instituto Mexicano del Seguro Social (IMSS) Siglo XXI. Autopsies and necropsy sampling for scientific use were authorized by a relative of the deceased patients. A total of 23 necropsy samples from both sexes (13 female, 10 male) were analyzed, with an average patient age of 56 years (range: 32–85 years). All donors died from heart failure or myocardial infarction. As an inclusion criterion, none of the deceased patients presented conditions characteristic of metabolic syndrome, such as obesity or elevated glucose levels. Autopsies were performed within the first 3 h after death. Pancreatic samples were fixed in 4% formalin/PBS for 12 h and processed using conventional histological techniques, followed by paraffin embedding. Sixteen necropsy samples (nine female and seven male) were used for IHC studies, and seven samples (four female and three male) were used for qRT-PCR analyses. All procedures adhered to the principles outlined in the Declaration of Helsinki (1964).

### Immunohistochemistry studies

Five micrometers of pancreatic sections were prepared for morphological studies. After antigen retrieval using citrate buffer and DIVA Declaker 10X (Cat. #DV2004MX, Biocare, U.S.A.; 1:10), at 120°C for 3 min, and endogenous peroxidase blocking solution (3%), the serial sections were incubated overnight at 4°C with polyclonal antibodies that recognize both human and mouse anti-Cx31.9 (Cat. #40-7400, Invitrogen, CA, U.S.A., 1:50) or anti-Cx36 (Cat. #36-4600, Invitrogen, CA, U.S.A.; 1:50) or mouse monoclonal anti-CK19 antibody (Cat. #SC-6278, Santa Cruz Biotechnology, Santa Cruz, CA, U.S.A.; 1:100) or the guinea pig anti-insulin (Cat. #PA1-26938, Invitrogen, CA, U.S.A.; 1:2000). After rinsing, sections were incubated for 1 h at 37°C with an HRP secondary antibody (Cat. #31460, Thermo Scientific, CA, U.S.A.; 1:500), and immunoreactivity was revealed by incubation in a 3,3'-diaminobenzidine (DAB) chromogen kit (Cat. #BDB2004Ll, Biocare Medical, U.S.A.) for 5 min. As negative controls, sections were incubated only with a secondary antibody. For each donor experiment, two sections were incubated with the anti-Cx31.9 antibody, one with the anti-insulin antibody, and a control section one without a primary antibody. Each experiment was done by triplicates to confirm the reproducibility of protein cell marker of interest detection.

### Densitometric diaminobenzidine quantification

Thirty-one images were acquired, including two controls without the primary antibody. Photomicrographs were captured under identical bright-field conditions using a Nikon Microphot FXA microscope with a 40× objective. Köhler illumination employed an Abbe condenser at three-fourths capacity and a light intensity of 9.3 (scale 4–12). Images were obtained with Nikon ACT-1 software at 3600 × 2880 resolution (10,368,000 pixels; 136 pixels/10 μm). Cx31.9 integrated density in μm^2^ (Cx31.9 ID) was calculated with a custom Python program [26] performing: (a) color deconvolution to separate HDX signals, (b) external-islet masking to discard non-islet signals, (c) internal-islet masking via normalization, histogram matching, and thresholding against the mean control DAB signal plus 1 standard deviation, followed by binarization to yield refined endocrine masks above background noise, and (d) application of the endocrine masks to the raw HDX images, which are subsequently globally normalized, resulting in a set of improved, segmented DAB signals from which the statistics are derived. Cx31.9 ID calculations were done in 31 images from several islets [2–6] from most of the 10 donors specimens used.

### RNA isolation and reverse transcription

Paraffin sections, 30–40 μm thick, were collected in sterile, RNase-free 1.5 ml Eppendorf tubes for total RNA isolation. RNA isolation, purification, and precipitation were performed using the MasterPure Complete DNA and RNA Purification kit (Cat. #MC85200, Epicenter, Madison, WI, U.S.A.) according to the manufacturer’s protocol. Finally, the RNA was resuspended in DEPC-treated water, and its concentration was measured using a spectrophotometer (NanoDrop 1000, Thermo Scientific, U.S.A.). RNA samples were stored at −80°C until further analysis. The purity of RNA from formalin-fixed, paraffin-embedded (FFPE) samples was assessed by quantifying the A260/280 ratio using a NanoDrop spectrophotometer (Thermo Fisher Scientific, U.S.A.). The observed range of this ratio, spanning from 1.80 to 1.89 (*n* = 7), was consistent with conventional RNA quality standards [27,28]. Furthermore, the preserved quality of FFPE cDNA was assessed by detecting ribosomal RNA bands 28S and 18S in a 2:1 ratio, as observed and quantified by agarose gel electrophoresis combined with densitometric analysis (ImageJ software package; National Institutes of Health, U.S.A.).

Reverse transcription (RT) of total RNA was performed using the High-Capacity cDNA Reverse Transcription Kit (Cat. #4368814, Applied Biosystems, U.S.A.) in a total volume of 20 μl. The mixture included 10 μl of RNA sample and 10 μl of 2× RT mix, which contained 2 μl of 10× RT buffer, 0.8 μl of 100 mM dNTPs, 2 μl of 10× RT random primers, 1 μl of MultiScribe^TM^ reverse transcriptase (5 U/μl), 1 μl of RNase inhibitor (2 U/μl), and 4.2 μl of nuclease-free H_2_O. Reactions were incubated at 37°C for 120 min and then stored at −20°C.

### Quantitative real-time PCR

The relative expression levels of the Cx31.9 and Cx36 genes were calculated using the 2^−ΔΔCt^ method [29]. Results were expressed as the fold change in gene expression, normalized to the housekeeping gene GAPDH. For this purpose, TaqMan gene expression assays (Cx31.9 Hs00987388_s1, Cx36 Hs00706940_s1, and GAPDH, Hs02786624_g1; Applied Biosystems) were used. cDNA from pancreatic tissue samples was screened in triplicate (see CT values summarized in the Supplementary Table S1) in a final volume of 10 μl, including 100 ng of cDNA template, 5 μl of 2× TaqMan Universal PCR Master Mix (Cat. #4304437, Applied Biosystems, U.S.A.), 0.5 μl of 20× TaqMan Gene Expression Assay, and 4.5 μl of RNase-free water. Negative controls included water instead of cDNA. Amplification was performed on a 7500 Real-Time PCR System (Applied Biosystems, Foster City, U.S.A.) under these conditions: initial activation at 50°C for 2 min and 95°C for 10 min, followed by 50 cycles of denaturation at 95°C for 30 s and annealing/extension at 65°C for 1 min. To visualize the expected amplified products, 10 μl of the PCR reactions were loaded onto a 2% agarose gel and stained with 0.5 mg/ml ethidium bromide.

### Statistical analysis

Statistical analyses of PCR experiments were conducted using GraphPad Prism version 8.0 (GraphPad Software, San Diego, CA, U.S.A.). Data are presented as mean ± standard deviation (SD). Group comparisons were performed with a two-tailed Student’s *t*-test, and a *P*-value < 0.05 was considered statistically significant.

## Results

**Figure 1:**
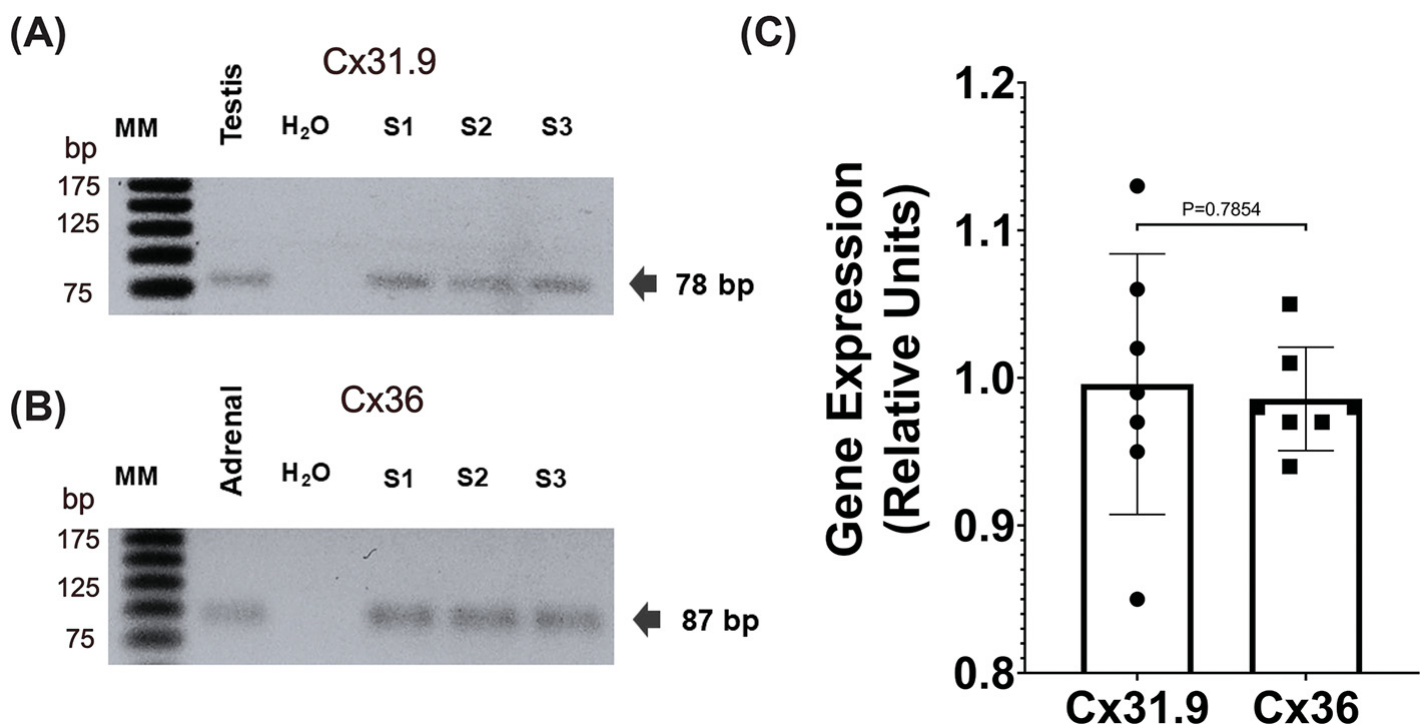
Expression of Cx31.9 mRNA in human pancreatic necropsy samples (**A**) Electrophoresis gel image showing 78 bp bands in lanes loaded with products from quantitative real-time PCR amplification using Cx31.9 oligonucleotides and cDNA reverse transcribed from total RNA from human autopsy pancreases (S1–S3) and testis paraffin sections, but not in control reactions with H_2_O as a substitute for cDNA as indicated. MM = molecular weight marker (75–175 bp); (**B**) Electrophoresis gel image showing 87 bp bands in lanes loaded with qRT-PCR reaction products obtained using Cx36 oligonucleotides and cDNA reverse transcribed from total RNA of paraffin sections of human pancreases (lanes 4–6) and adrenal gland (lane 2) autopsy samples, but not in a lane loaded with a reaction where H_2_O replaces cDNA, as indicated, (**C**) Scatter dot plot graph with normalized mean levels of Cx31.9/GAPDH and Cx36/GAPDH mRNA levels from *n* = 7 pancreases analyzed. Data are expressed as mean ± SD. Statistical analysis was performed using an unpaired two-tailed Student’s *t*-test, showing no significant differences between groups (*P* = 0.7854).

To determine whether GJD3 [24] mRNA is expressed in the human pancreas, PCR reactions were performed using cDNA synthesized from total RNA isolated from paraffin-embedded pancreatic tissue from four female and three males, with oligonucleotides specific for Cx31.9. Figure 1 shows that Cx31.9 mRNA was detected in all donor samples, with expression levels comparable to those of Cx36 (*P* = 0.7854). Figure 1A shows an electrophoresis gel image in which a band of the expected molecular weight for Cx31.9 (78 bp) is identified in lanes loaded with reactions obtained from human pancreases (S1–S3) and human testis, used as a positive control, but not in reactions where H_2_O replaces cDNA. Figure 1B shows an image of another electrophoresis gel, where a band of 87 bp, as expected for the Cx36 amplicon, is identified in lanes loaded with qRT-PCR reactions from human pancreases (S1–S3), as well as from the human adrenal gland, used as positive control, but not in the lane without cDNA. Figure 1C presents a bar chart illustrating a scatter plot showing that normalized mean Cx31.9/GAPDH and Cx36/GAPDH mRNA levels are similar across different pancreatic donor samples.

**Figure 2:**
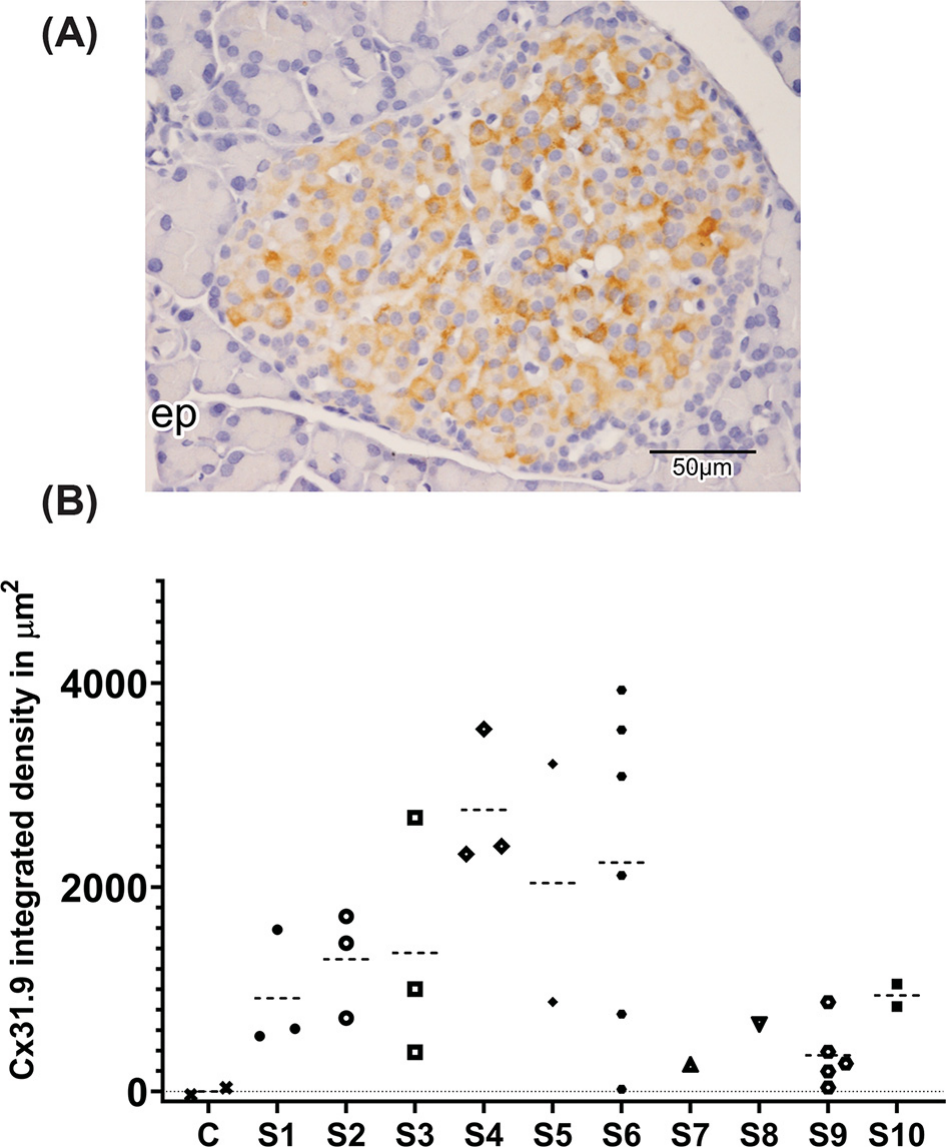
Connexin 31.9 is expressed in most endocrine islet cells in the human pancreas (**A**) Image of a paraffin-embedded section of the human pancreas incubated with anti-Cx31.9 and revealed with DAB, where anti-Cx31.9-positive staining is localized in cells of a large islet (i) within the exocrine pancreas (ep). Heterogeneity in anti-Cx31.9 staining was observed within a single islet. (**B**) Distribution of Cx31.9 integrated density in μm^2^ (Cx31.9 ID) values calculated from 31 analyzed islet images from 10 different donors (S1–S10) using a custom-designed algorithm (see the ‘Materials and methods’ section). Background noise from the DAB signal was calculated as the mean + 1 SD, using islet images of two control donors (C) not exposed to the primary antibody. This number was subtracted from each islet Cx31.9 ID value. Each symbol corresponds to a Cx31.9 ID value calculated for a single islet. Cx31.9 ID for different donors is represented by different symbols. Dashed lines represent the average Cx31.9 obtained from the islets of individual donors. The median value for all donor samples was 1324.96, with a minimum of 19.97 and a maximum of 3931.5.

To determine whether the GJD3-coded protein is expressed in human islets, pancreatic sections from sixteen donor autopsy specimens, nine female and seven men, were incubated with a specific anti-Cx31.9 antibody and analyzed by IHC. Figure 2 shows an image of a large islet (A) in which anti-Cx31.9 staining is positive in most of its cells. A similar cell distribution pattern of anti-Cx31.9 protein staining was detected in most donor samples analyzed (15 out of 16). Staining intensity varies within cells of a single islet (Figure 2A) and among islets from a single donor as well as between different donor specimens. Variation among these last ones is shown in Figure 2B, which illustrates the distribution of the Cx31.9 integrated density in a μm^2^ (Cx31.9 ID) values calculated for 31 islet images from ten donors each indicated with a different symbol. The median of Cx31.9 ID for all donor samples was 1324.96, with a minimum of 19.97 and a maximum of 3931.5. The islet’s Cx31.9-DAB staining area varies from 969.9 to 14,020 μm^2^. These findings indicate that the Cx31.9 protein is expressed in human pancreatic beta cells, which are the most abundant cell type in the endocrine islets.

**Figure 3:**
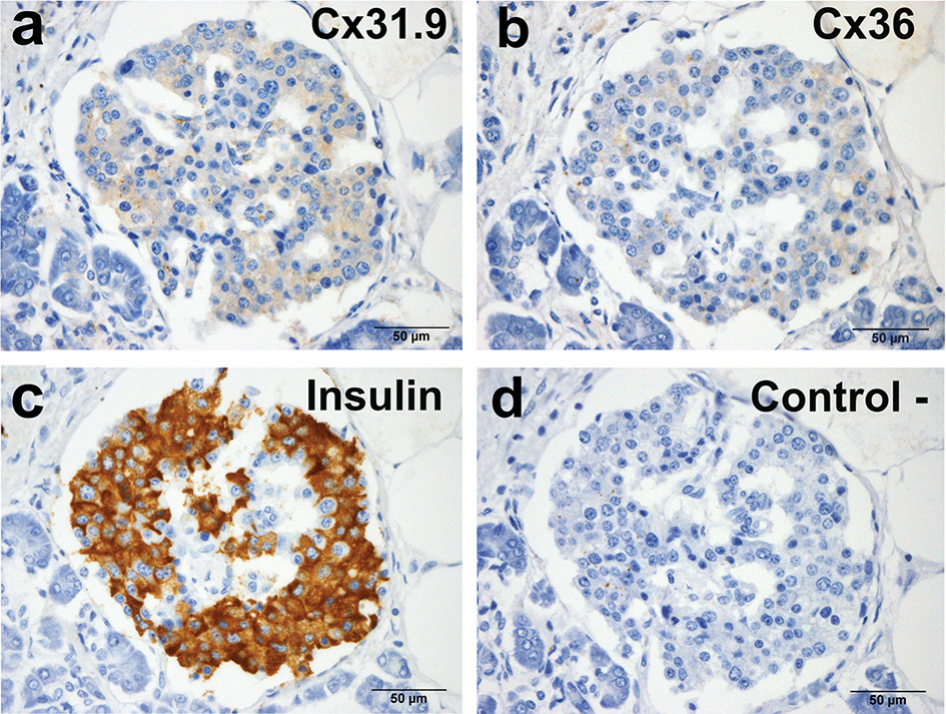
Connexin 31.9 is expressed in human pancreatic beta cells Images show paraffin-embedded serial sections of a human pancreas stained with anti-Cx31.9, anti-Cx36, or anti-insulin antibodies. Inner islet cells were positively stained with anti-Cx31.9 (**a**), showing a cell distribution similar to that labeled with anti-Cx36 (**b**) or anti-insulin (**c**) antibodies, as shown in serial sections. However, no staining was observed in a serial section incubated only with the secondary antibody. (**d**) Scale bars are included in the figures.

To gather additional evidence for Cx31.9 localization in pancreatic beta cells, human pancreatic serial sections from five autopsy samples were stained with specific anti-Cx31.9, anti-Cx36, or anti-insulin antibodies. Figure 3 shows representative IHC images in which anti-Cx31.9 staining (a) was localized to the inner cell mass of the islet, a region that was also positively stained with anti-Cx36 (b) or with anti-insulin (c) in serial sections, but not in a serial section incubated only with the secondary antibody (d). These findings further support that beta cells express the Cx31.9 protein.

**Figure 4:**
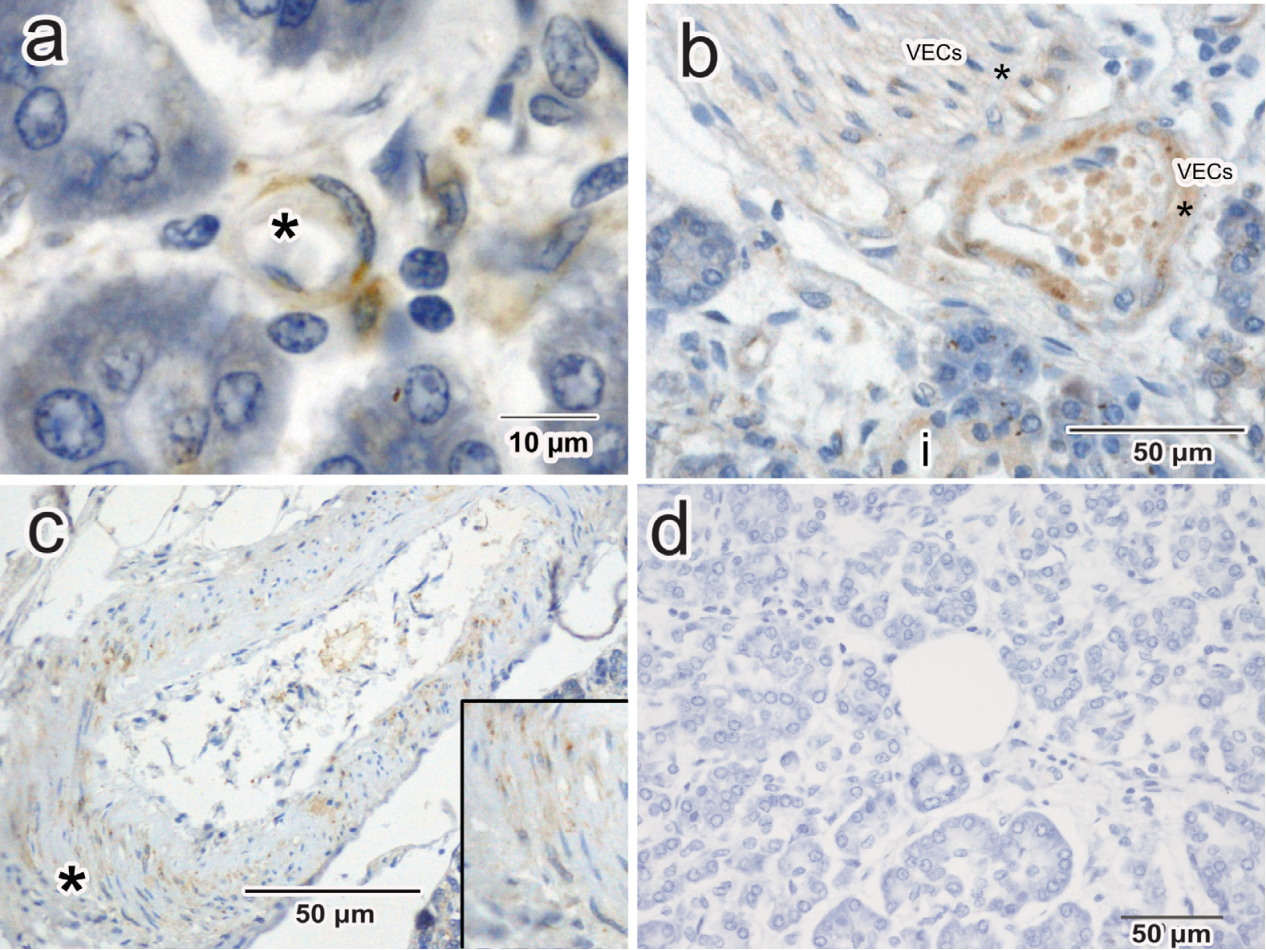
Endothelial cells in blood vessels of all calibers express the protein Cx31.9 Images of human pancreatic sections incubated with anti-Cx31.9 and revealed by IHC with DAB. Anti-Cx31.9-DAB staining is localized in VECs of large (**a**), medium (**b**), and small (**c**) diameter blood vessels, but not in vessels incubated only with the secondary antibody (**d**). In panel (b), in the neighborhood of the medium diameter vessel, some islet cells (i) are indicated. Scale bars are shown in each panel.

To determine whether the GJD3-coded protein is expressed in the exocrine human pancreas, the exocrine regions were analyzed in donor sections where islets were found to be positively stained with the anti-Cx31.9 antibody and used as a positive internal control. Figure 4 shows a representative image of a pancreatic section in which Cx31.9-positive staining is localized to vascular endothelial cells (VECs) of large (Figure 4a), medium (Figure 4b), and small-caliber (Figure 4c) blood vessels, but not in sections incubated only with the secondary antibody (Figure 4d). These findings suggest that, as in mice [22], human VECs express the Cx31.9 gap junction protein.

**Figure 5:**
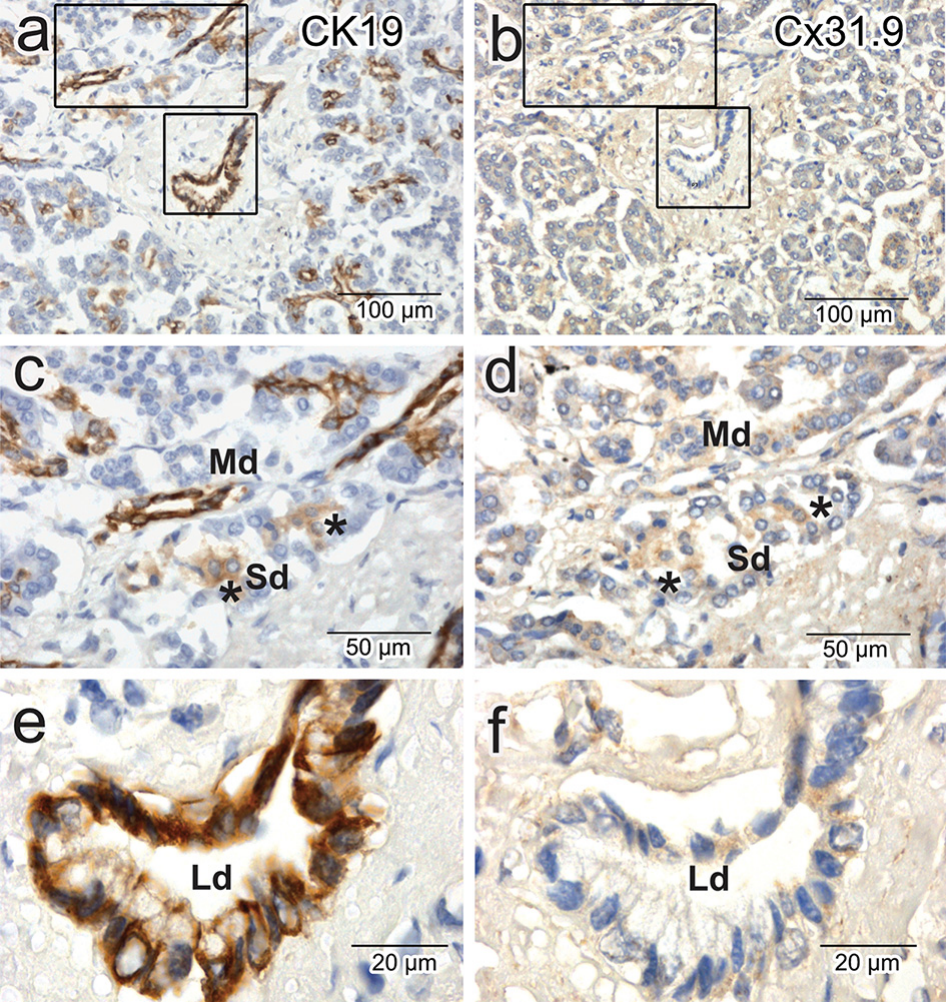
Connexin 31.9 is expressed in human pancreatic ductal epithelial cells Images of serial pancreatic sections stained with anti-CK19 (**a, c, e**) or anti-Cx31.9 (**b, d, f**) and revealed by IHC. The upper panels show inner ductal epithelial cells, identified by positive staining with anti-CK19 (**a**), which are also stained with anti-Cx31.9 (**b**) in large (Ld), medium (Md), and small-diameter ducts (Sd; asterisks). Amplifications of the two boxed areas are shown in the corresponding medium and lower panels to better identify Cx31.9 staining across the various ductal categories. Each panel includes its scale bar.

Likewise, to determine whether Cx31.9 protein is expressed in human ductal epithelial cells, pancreatic serial sections from five donors were incubated with an antibody against cytokeratin 19 (CK19), a marker of ductal epithelial cells [30], and with an anti-Cx31.9 antibody. Figure 5 shows that epithelial cells from ducts of different diameters were positively stained with anti-CK19 and anti-Cx31.9. The regions boxed in the upper panels (a, b) show different ductal categories, which are shown at higher magnification in the middle (c, d) and lower (e, f) panels to better visualize the distribution of Cx31.9 staining in epithelial cells of small-diameter (c and d; Sd, asterisks), medium-diameter (c, d; Md), and large-diameter (e and f; Ld) ducts, as indicated.

## Discussion

In the present study, using IHC with a previously characterized anti-Cx31.9 antibody, we provide the first evidence that the Cx31.9 protein is expressed in the human pancreas. We show that Cx31.9 protein staining is localized to pancreatic islet beta cells, as well as to pancreatic exocrine ductal epithelial and vascular endothelial cells. Additionally, using qRT-PCR, we provide evidence that in the human pancreas, the mean normalized levels of Cx31.9 and Cx36 mRNAs are balanced.

The antibody used in the present study specifically labels Cx31.9 in human tissues, as shown by Western blot and IHC studies in the testis conducted by the manufacturer and others [31]. It also labels mouse pancreatic islets [20,22]. Here, using the same antibody in human pancreatic sections with IHC, we demonstrate that the cellular distribution of Cx31.9 in human islet beta cells, vascular endothelial cells, and ductal epithelial cells is similar to that described for Cx30.2 in mouse pancreatic islet beta cells [20] and in exocrine ductal epithelium and vascular endothelial cells [22]. The identification of these two orthologous proteins by the same antibody (see the ‘Materials and methods’ section) results from the high sequence homology between Cx31.9 and Cx30.2 (79%) [32].

Most islet cells tested positive for anti-Cx31.9, although staining intensity varied within individual cells from a single islet (Figure 2A) and between islets from the same or different donors, as shown by the diverse Cx31.9 ID calculated across islets from ten donor samples analyzed. These findings, along with the similar Cx31.9 cell distribution pattern observed in all islets analyzed, strongly support the conclusion that this connexin is expressed in human pancreatic islets.

While the number of donor samples was limited in the present study, a consistent islet cell distribution of Cx31.9 staining pattern across all analyzed samples revealed that most islet cells were stained. Our results showed that Cx31.9 staining intensity varied among single islet beta cells, islets from different donors, and within islets from a single donor. This was indicated by the noticeable variation in the calculated DAB density per islet among the donor section samples. These differences in density levels may result from intrinsic variations in the expression of the Cx31.9 protein. This hypothesis is consistent with the heterogeneity in the levels of expression of other proteins and metabolic enzymes previously described within beta cells in a single islet as well as among islets within a single pancreas [33,34]. The latter is supported by the finding of these differences also within islets from a single donor. Nonetheless, differences in Cx31.9 ID among islets from different donors may also be influenced by slight variations in sample processing and preservation. The latter is the most likely explanation for the single-donor sample with negative staining observed in our study.

Insulin-secreting cells are the most abundant islet cell population in both rodents and humans. Thus, the widespread anti-Cx31.9 staining observed in most islet cells indicates that this connexin is expressed in beta cells. This cell-type distribution was strongly supported by serial section analysis, in which anti-Cx31.9-, anti-Cx36-, and anti-insulin-positive stained cells showed similar localization. However, we cannot rule out the possibility that other human endocrine cells in the islet also express Cx31.9. In addition, not all peripheral or central islet cells were stained, which may be due to relatively low Cx31.9 expression falling below the DAB detection threshold or to the absence of this connexin subtype in other endocrine cell types. In humans, a larger proportion of glucagon-secreting cells is found within the inner islet mass, in contrast with rodents, where most glucagon-secreting cells are localized toward the islet periphery. Cx30.2 does not localize to glucagon cells in rodents [22]; nonetheless, in humans, further immunofluorescent and electron microscopic studies are necessary to determine whether Cx31.9 is expressed in glucagon and other endocrine islet cells.

In mouse pancreatic sections, anti-Cx30.2 labeling and anti-Cx36 immunofluorescent dots were co-localized at regions of cell membrane apposition [20], suggesting their close proximity within the same gap junction plaques. Intercellular dye transfer has been observed in human pancreatic islet cells and is believed to result from beta-cell-to-beta-cell communication via Cx36 channels [35]. However, because transfection of Cx31.9 cDNA into connexin-deficient cell systems results in the formation of gap junction channels [36], and the GJD3-coding protein is expressed in human islet beta cells as shown here, these findings suggest that Cx31.9 may play a role in direct intercellular communication in this cell type. In the present study, we did not attempt double immunofluorescence incubations because human necropsy sections were formalin-fixed and paraffin-embedded, a process known to increase background fluorescence and reduce the signal-to-noise ratio for detection. Co-incubation of human pancreatic sections with immunogold antibodies might determine whether Cx31.9 is localized to the membrane of pancreatic beta cells and/or to gap junction channels.

IHC studies shown here also revealed that anti-Cx31.9 staining is localized to human pancreatic endothelial cells in blood vessels of all diameters. This cell distribution is consistent with the localization of Cx30.2 in rodent pancreatic endocrine [20] and exocrine [22] endothelial blood vessels as well as in rat retinal endothelial cells [21]. Interestingly, cultured retinal endothelial cells exposed to a high glucose concentration (30 mM) down-regulate their expression [21].

Likewise, we document here that Cx31.9 is expressed in human epithelial cells from different duct categories. This was revealed in serial sections, which showed that both anti-Cx31.9- and anti-CK19-positive cells had the same distribution. These findings are consistent with previous findings from our group, which demonstrated that Cx30.2 is localized in epithelial ductal cells in mice, rats, and guinea pigs [22]. Further ultrastructural studies are needed to determine whether Cx31.9 localizes to the cell membrane and/or gap junctions in these human pancreatic cell types.

The similar normalized mean Cx31.9 and Cx36 mRNA levels observed across all donor samples analyzed support their balanced abundance in human islet beta cells. This finding is consistent with the localization of Cx31.9 staining in most islet cells across most donor samples in our IHC studies. The identification of GJD3 RNA expression shown here is consistent with previous studies, in which a band of the expected size for Cx31.9 mRNA was found in total human pancreas preparations by Northern blot analysis [24], as well as in human islets, using multi-Cx analysis by endpoint PCR studies and SYBR Green probes [25].

In conclusion, the IHC studies presented here document that the Cx31.9 protein is expressed in both the endocrine and exocrine human pancreas in both sexes. Specifically, Cx31.9 is expressed in human pancreatic beta cells, as indicated by widespread anti-Cx31.9 staining throughout most islet cells and by the detection of a similar distribution of anti-Cx31.9, anti-Cx36, and anti-insulin staining in serial sections across islet cells from different donor samples. Together with the balanced, normalized Cx31.9 and Cx36 mRNA levels shown here, these findings support the relevance of the Cx31.9 protein to human islet beta-cell physiology.

Likewise, IHC studies shown here documented anti-Cx31.9 staining in exocrine vascular endothelial cells and epithelial ductal cells.

The cell distribution patterns of Cx31.9 protein expression in human islet beta cells, exocrine vascular endothelial cells, and epithelial ductal cells are shared across the pancreases of different rodent species, supporting the functional role of this GJD3 protein in both human endocrine and exocrine cell pathophysiology and warranting further study.

## Open Access

Open access for this article was enabled by the participation of Universidad Nacional Autónoma de México (UNAM) in an all-inclusive Read & Publish agreement with Portland Press and the Biochemical Society. The authors agree to publish the manuscript under open access terms.

## Abbreviations

Cx30.2: connexin 30.2
Cx36: connexin 36
Cxs: connexins
CK19: cytokeratin 19
DAB: 3,3′-diaminobenzidine
VECs: vascular endothelial cells
FFPE: formalin-fixed, paraffin-embedded
IHC: immunohistochemistry
IMSS: Instituto Mexicano del Seguro Social
RT: reverse transcription
qRT-PCR: quantitative reverse transcription polymerase chain reaction
SD: standard deviation

## References

[1] International Diabetes Federation, About the International Diabetes Federation [Internet]. Brussels (Belgium): International Diabetes Federation[cited 2025 Jun 4]. Available from: https://idf.org/our-activities/about-us/

[2] Michon L., Nlend Nlend R., Bavamian S., Bischoff L., Boucard N., Caille D. et al. (2005) Involvement of gap junctional communication in secretion. Biochim. Biophys. Acta Biomembr. 1719, 82–101 10.1016/j.bbamem.2005.11.003 16359942

[3] Pérez-Armendariz E.M. (2013) Connexin 36, a key element in pancreatic beta cell function. Neuropharmacology 75, 557–566 10.1016/j.neuropharm.2013.08.015 23973309

[4] Pérez‐Armendariz E.M., Luna J., Aceves C. and Tápia D. (1995) Connexins 26, 32 and 43 are expressed in virgin, pregnant and lactating mammary glands. Dev. Growth Differ. 37, 421–431 10.1046/j.1440-169X.1995.t01-3-00009.x 37281114

[5] Lucaciu S.A., Leighton S.E., Hauser A., Yee R. and Laird D.W. (2023) Diversity in connexin biology. J. Biol. Chem. 299, 105263 10.1016/j.jbc.2023.105263 37734551 PMC10598745

[6] Srinivas M., Verselis V.K. and White T.W. (2017) Human diseases associated with connexin mutations. Biochim. Biophys. Acta 1860, 192 10.1016/j.bbamem.2017.04.024 PMC5659969 28457858

[7] Moreno A.P., Berthoud V.M., Pérez-Palacios G. and Pérez-Armendariz E.M. (2005) Biophysical evidence that connexin-36 forms functional gap junction channels between pancreatic mouse beta-cells. Am. J. Physiol. Endocrinol. Metab. 288, E948 10.1152/ajpendo.00216.2004 15625088

[8] Pérez-Armendariz E.M., Roy C., Spray D.C. and Bennett M.V. (1991) Biophysical properties of gap junctions between freshly dispersed pairs of mouse pancreatic beta cells. Biophys. J. 59, 76–92 10.1016/S0006-3495(91)82200-7 2015391 PMC1281120

[9] Srinivas M., Rozental R., Kojima T., Dermietzel R., Mehler M., Condorelli D.F. et al. (1999) Functional properties of channels formed by the neuronal gap junction protein connexin36. J. Neurosci. 19, 9848–9855 10.1523/JNEUROSCI.19-22-09848.1999 10559394 PMC6782942

[10] Allagnat F., Martin D., Condorelli D.F., Waeber G. and Haefliger J.A. (2005) Glucose represses connexin36 in insulin-secreting cells. J. Cell Sci. 118, 5335–5344 10.1242/jcs.02600 16263767

[11] Serre-Beinier V., Le Gurun S., Belluardo N., Trovato-Salinaro A., Charollais A., Haefliger J.A. et al. (2000) Cx36 preferentially connects beta-cells within pancreatic islets. Diabetes 49, 727–734 10.2337/diabetes.49.5.727 10905480

[12] Klee P., Allagnat F., Pontes H., Cederroth M., Charollais A., Caille D. et al. (2011) Connexins protect mouse pancreatic β cells against apoptosis. J. Clin. Invest. 121, 4870–4879 10.1172/JCI40509 22056383 PMC3225984

[13] Ravier M.A., Güldenagel M., Charollais A., Gjinovci A., Caille D., Söhl G. et al. (2005) Loss of connexin36 channels alters beta-cell coupling, islet synchronization of glucose-induced Ca2+ and insulin oscillations, and basal insulin release. Diabetes 54, 1798–1807 10.2337/diabetes.54.6.1798 15919802

[14] Head W.S., Orseth M.L., Nunemaker C.S., Satin L.S., Piston D.W. and Benninger R.K.P. (2012) Connexin-36 gap junctions regulate *in vivo* first- and second-phase insulin secretion dynamics and glucose tolerance in the conscious mouse. Diabetes 61, 1700–1707 10.2337/db11-1312 22511206 PMC3379660

[15] Carvalho C.P.F., Oliveira R.B., Britan A., Santos-Silva J.C., Boschero A.C., Meda P. et al. (2012) Impaired β-cell-β-cell coupling mediated by Cx36 gap junctions in prediabetic mice. Am. J. Physiol. Endocrinol. Metab. 303, 144–151 10.1152/ajpendo.00489.2011 22569071

[16] Bosco D., Haefliger J.A. and Meda P. (2011) Connexins: key mediators of endocrine function. Physiol. Rev. 91, 1393–1445 10.1152/physrev.00027.2010 22013215

[17] Farnsworth N.L. and Benninger R.K.P. (2014) New insights into the role of connexins in pancreatic islet function and diabetes. FEBS Lett. 588, 1278 10.1016/j.febslet.2014.02.035 24583073 PMC4004767

[18] Bukauskas F.F., Kreuzberg M.M., Rackauskas M., Bukauskiene A., Bennett M.V.L., Verselis V.K. et al. (2006) Properties of mouse connexin 30.2 and human connexin 31.9 hemichannels: Implications for atrioventricular conduction in the heart. Proc. Natl. Acad. Sci. U.S.A. 103, 9726–9731 10.1073/pnas.0603372103 16772377 PMC1480474

[19] Teubner B., Degen J., Söhl G., Güldenagel M., Bukauskas F.F., Trexler E.B. et al. (2000) Functional expression of the murine connexin 36 gene coding for a neuron-specific gap junctional protein. J. Membr. Biol. 176, 249 10.1007/s002320001094 10931976 PMC3659790

[20] Coronel-Cruz C., Hernández-Tellez B., López-Vancell R., López-Vidal Y., Berumen J., Castell A. et al. (2013) Connexin 30.2 is expressed in mouse pancreatic beta cells. Biochem. Biophys. Res. Commun. 438, 772–777 10.1016/j.bbrc.2013.06.100 23831630

[21] Manasson J., Tien T., Moore C., Kumar N.M. and Roy S. (2013) High glucose-induced downregulation of connexin 30.2 promotes retinal vascular lesions: implications for diabetic retinopathy. Invest. Ophthalmol. Vis. Sci. 54, 2361–2366 10.1167/iovs.12-10815 23385797 PMC3626527

[22] Coronel-Cruz C., Sánchez I., Hernández-Tellez B., Rodríguez-Mata V., Pinzón-Estrada E., Castell-Rodríguez A. et al. (2018) Connexin 30.2 is expressed in exocrine vascular endothelial and ductal epithelial cells throughout pancreatic postnatal development. Acta Histochem. 120, 558–565 10.1016/j.acthis.2018.06.007 30100173

[23] Gosak M., Yan-Do R., Lin H., Macdonald P.E. and Stozer A. (2022) Ca^2+^ oscillations, waves, and networks in islets from human donors with and without type 2 diabetes. Diabetes 71, 2584–2596 10.2337/db22-0004 36084321 PMC9750953

[24] Nielsen P.A., Beahm D.L., Giepmans B.N.G., Baruch A., Hall J.E. and Kumar N.M. (2002) Molecular cloning, functional expression, and tissue distribution of a novel human gap junction-forming protein, connexin-31.9: interaction with ZONA occludens protein-1. J. Biol. Chem. 277, 38272–38283 10.1074/jbc.M205348200 12154091

[25] Serre-Beinier V., Bosco D., Zulianello L., Charollais A., Caille D., Charpantier E. et al. (2009) Cx36 makes channels coupling human pancreatic β-cells, and correlates with insulin expression. Hum. Mol. Genet. 18, 428–439 10.1093/hmg/ddn370 19000992 PMC2638800

[26] Page-Pérez E.R. (2026) Python Program. IHC Protein Staining Analysis. Available from: https://github.com/EnriquePagePerez/IHC-Protein-Staining-Analysis, Last accessed: 9 de Junio de 2026

[27] Bustin S.A., Benes V., Garson J.A., Hellemans J., Huggett J., Kubista M. et al. (2009) The MIQE guidelines: minimum information for publication of quantitative real-time PCR experiments. Clin. Chem. 55, 611–622 10.1373/clinchem.2008.112797 19246619

[28] Lehmann U. and Kreipe H. (2001) Real-time PCR analysis of DNA and RNA extracted from formalin-fixed and paraffin-embedded biopsies. Methods 25, 409–418 10.1006/meth.2001.1263 11846610

[29] Pfaffl M.W. (2001) A new mathematical model for relative quantification in real-time RT-PCR. Nucleic Acids Res. 29, E45 10.1093/nar/29.9.e45 11328886 PMC55695

[30] Martin-Pagola A., Sisino G., Allende G., Dominguez-Bendala J., Gianani R., Reijonen H. et al. (2008) Insulin expression and proliferation in ductal cells in the transplanted pancreas of patients with type 1 diabetes and recurrence of autoimmunity. Diabetologia 51, 1803 10.1007/s00125-008-1105-x 18696047 PMC3019613

[31] Pelletier R.M., Layeghkhavidaki H., Kumar N.M. and Vitale M.L. (2020) Cx30.2 deletion causes imbalances in testicular Cx43, Cx46, and Cx50 and insulin receptors. Reciprocally, diabetes/obesity alters Cx30.2 in mouse testis. Am. J. Physiol. Regul. Integr. Comp. Physiol. 318, R1078–R1090 10.1152/ajpregu.00044.2020 32348681 PMC7311678

[32] Nielsen P.A. and Kumar N.M. (2003) Differences in expression patterns between mouse connexin-30.2 (Cx30.2) and its putative human orthologue, connexin-31.9. FEBS Lett. 540, 151–156 10.1016/S0014-5793(03)00252-7 12681499

[33] Duh M., Šterk M., Križančić Bombek L., MacDonald P.E., Stožer A. and Gosak M. (2025) Spatially bound functional heterogeneity drives modular organization in β-cell networks. Biophys. J. 124, 3008–3022 10.1016/j.bpj.2025.07.043 40765144 PMC12709238

[34] Stefan Y., Meda P., Neufeld M. and Orci L. (1987) Stimulation of insulin secretion reveals heterogeneity of pancreatic B cells *in vivo*. J. Clin. Invest. 80, 175–183 10.1172/JCI113045 3110211 PMC442216

[35] Charpantier E., Cancela J. and Meda P. (2007) Beta cells preferentially exchange cationic molecules via connexin 36 gap junction channels. Diabetologia 50, 2332–2341 10.1007/s00125-007-0807-9 17828386

[36] White T.W., Srinivas M., Ripps H., Trovato-Salinaro A., Condorelli D.F. and Bruzzone R. (2002) Virtual cloning, functional expression, and gating analysis of human connexin31.9. Am. J. Physiol. Cell Physiol. 283, C960–C970 10.1152/ajpcell.00163.2002 12176752

